# Supplementary material for: An extended substrate screening strategy enabling a low lattice mismatch for highly reversible zinc anodes
Source: Nat Commun. 2024 Jan 25;15:753. doi: 10.1038/s41467-024-44893-0 (PMC10810881; doi:10.1038/s41467-024-44893-0)
Supplement: Supplementary file 1 — Supplementary Information [file 41467_2024_44893_MOESM1_ESM.pdf]

Supplementary Information

An extended substrate screening strategy enabling a low lattice mismatch for highly reversible Zn anodes

Zhiyang Zheng[1,#], Xiongwei Zhong[1,#], Qi Zhang[1,#], Mengtian Zhang[1], Lixin Dai[1], Xiao Xiao[1], Jiahe Xu[1], Miaolun Jiao[1], Boran Wang[1], Hong Li[1], Yeyang Jia[1], Rui Mao[1], Guangmin Zhou[1,*]

[1] *Tsinghua-Berkeley Shenzhen Institute & Tsinghua Shenzhen International Graduate School, Tsinghua University, Shenzhen 518055, China*

* Corresponding authors: Guangmin Zhou (Email: guangminzhou@sz.tsinghua.edu.cn)

[#] These authors contributed equally to this work.

This file includes:

Supplementary Text

Supplementary Fig. 1 to Fig. 29

Supplementary Table 1 to Table 8

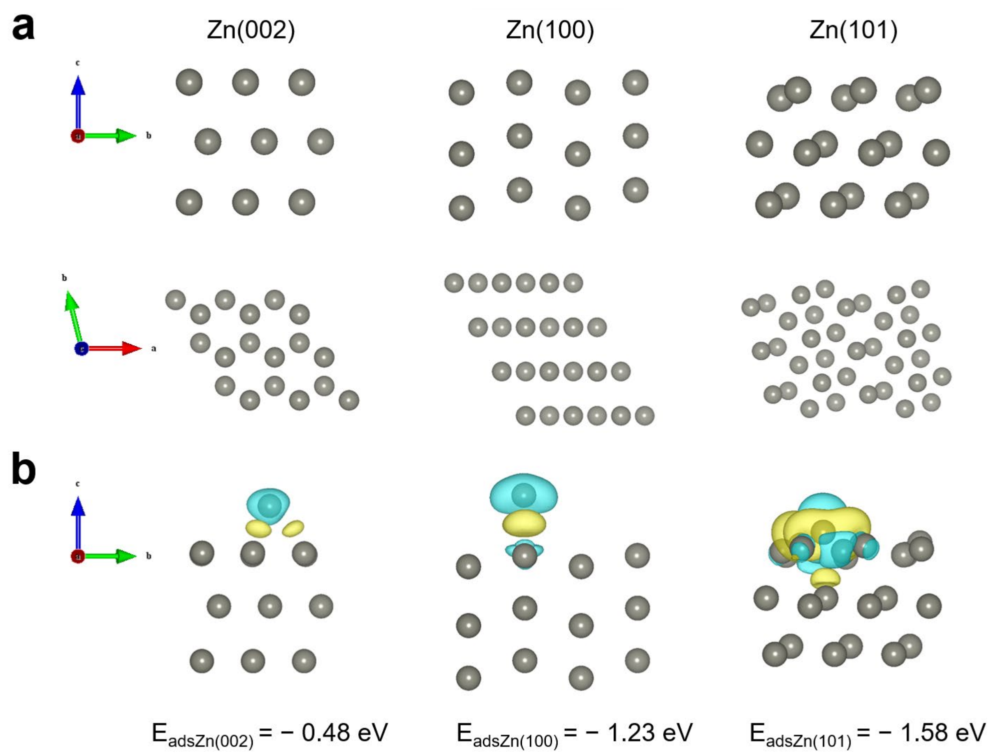

Supplementary Fig. 1 Atomic arrangements and calculated Zn adsorption energy of different crystalline planes of zinc. a Atomic arrangements of Zn(002) plane, Zn(100) plane and Zn(101) plane, respectively. **b** Calculated Zn adsorption energy on the surface of Zn(002) plane, Zn(100) plane and Zn(101) plane, respectively.

Supplementary Note 1. Based on the calculation results, Zn adsorption energy on the surface of Zn(002) plane is much lower than that of Zn(100) plane and Zn(101) plane, which means that the preferential order of Zn atoms deposited on the three planes is as follows: Zn(101) > Zn(100) > Zn(002). Therefore, to induce the growth of Zn deposited on the Zn(002) plane, it is essential to use a substrate that has a high level of compatibility with the Zn(002) lattice plane.

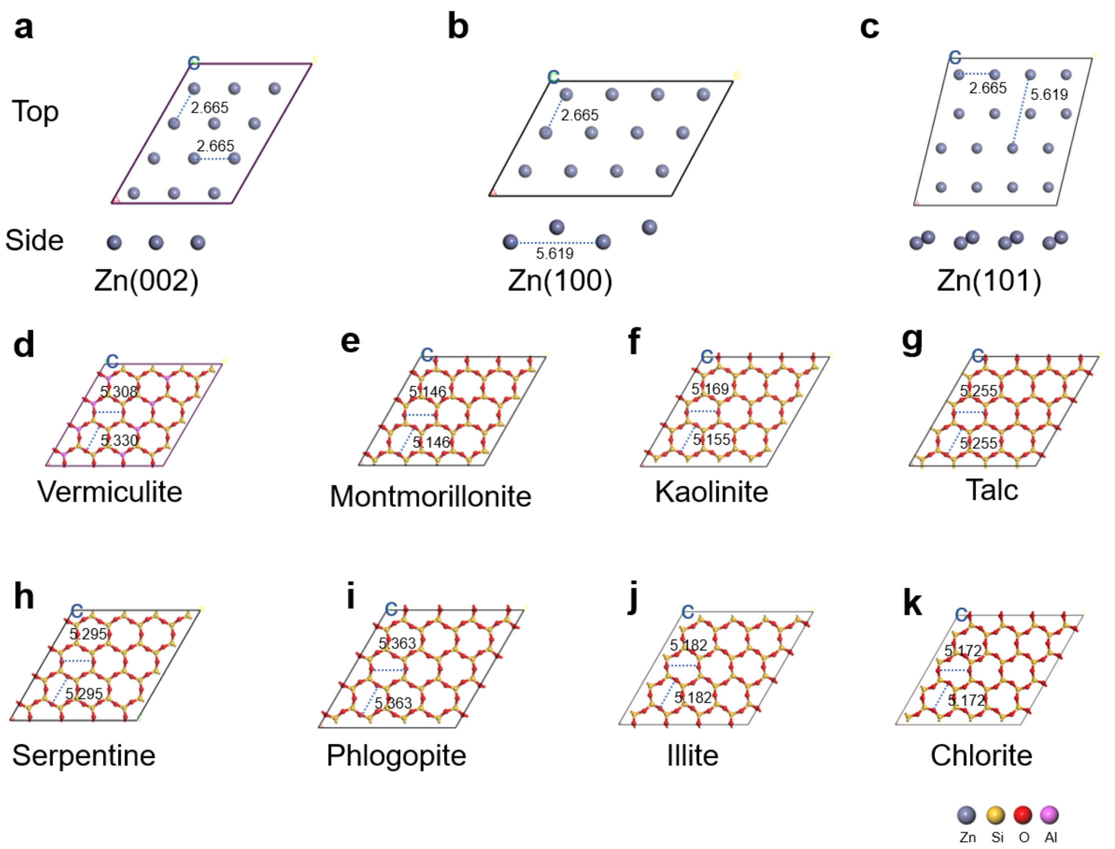

Supplementary Fig. 2 Atomic arrangements and crystal lattice of Zn metal and eight clay materials. Atomic arrangements of different crystalline planes for Zn metal, including Zn (002) plane (**a**), Zn (100) plane (**b**) and Zn (101) plane (**c**). Atomic arrangements of typical 2D clay systems, including vermiculite (**d**), montmorillonite (**e**), kaolinite (**f**), talc (**g**), serpentine (**h**), phlogopite (**i**), illite (**j**), chlorite (**k**). The crystal lattice of 2D clay materials was produced with the assistance of Materials Project and VESTA software.

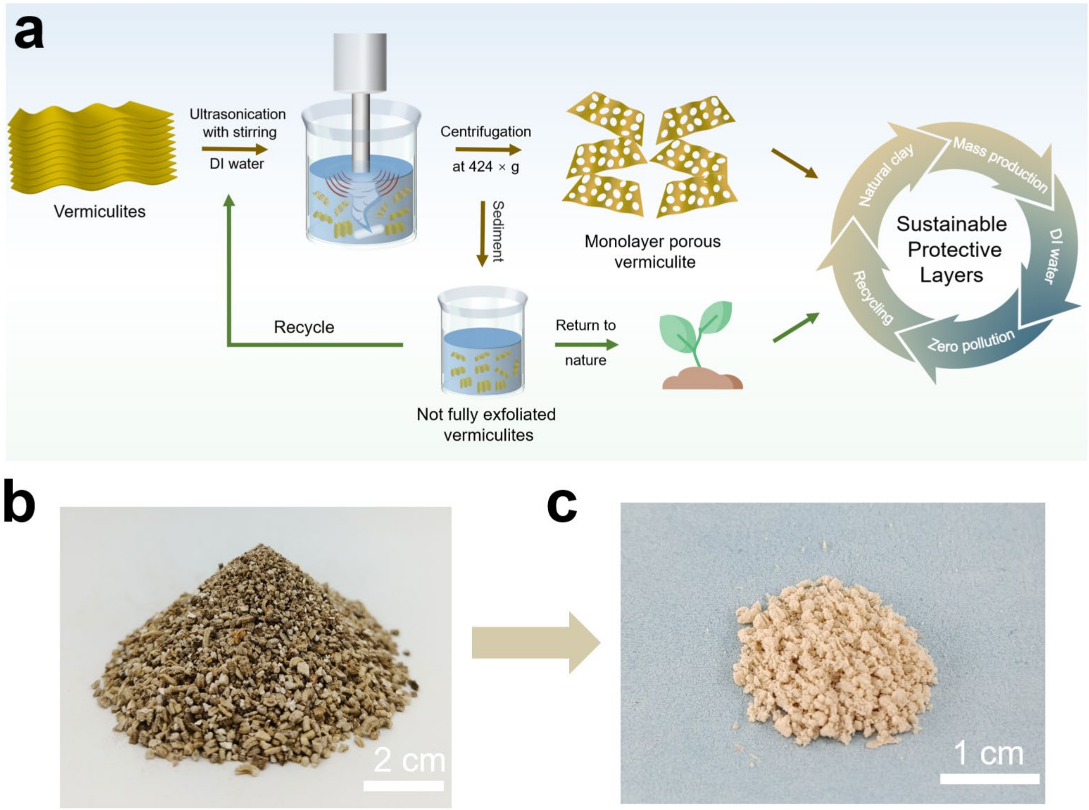

Supplementary Fig. 3 a Schematic of the preparation of MPVMTs. The pictures of typical VMT raw minerals **(b)** and MPVMT powders **(c)** after freeze-drying.

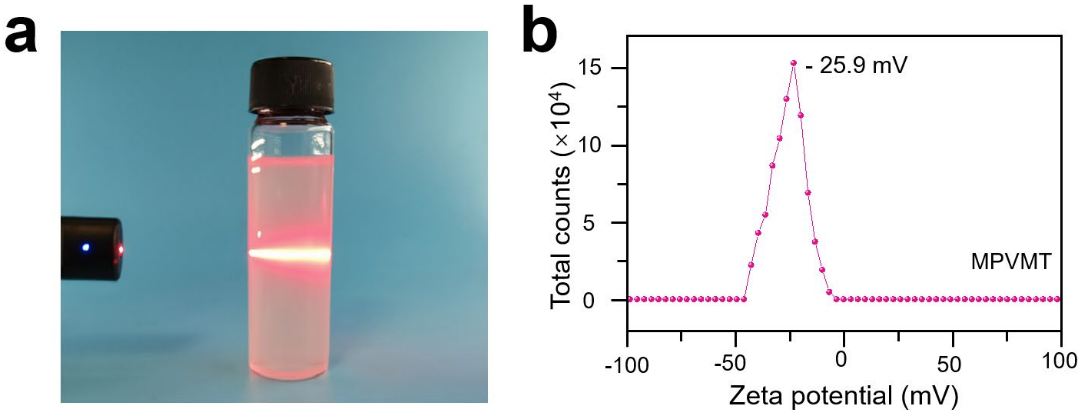

Supplementary Fig. 4 Characterizations of MPVMT dispersions. a Tyndall effect of MPVMT dispersions, confirming their colloidal state. **b** Zeta potential (−25.9 mV) of a MPVMT dispersion.

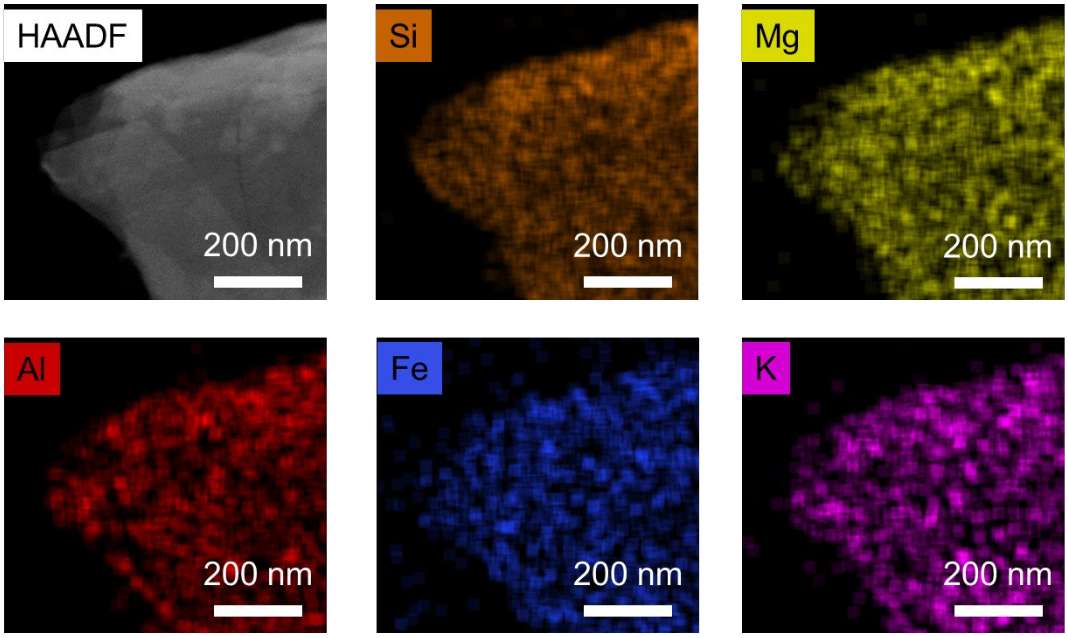

Supplementary Fig. 5 HAADF-STEM micrograph of MPVMT and corresponding EDS elemental mapping.

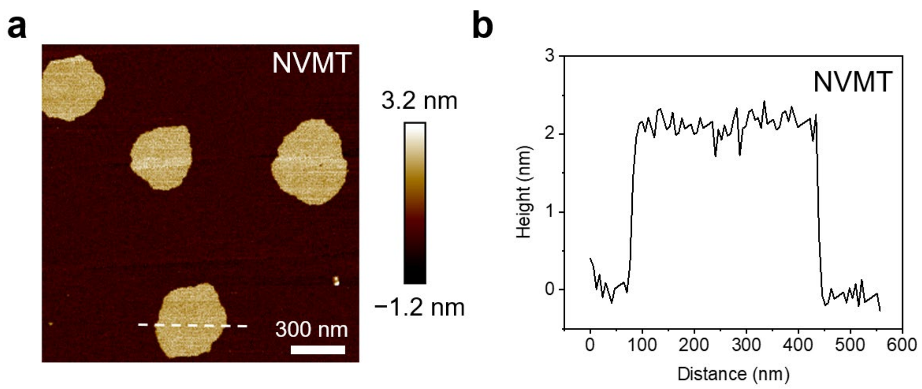

Supplementary Fig. 6 a AFM image of exfoliated monolayer NVMT. **b** The height profile of the corresponding lines of NVMT.

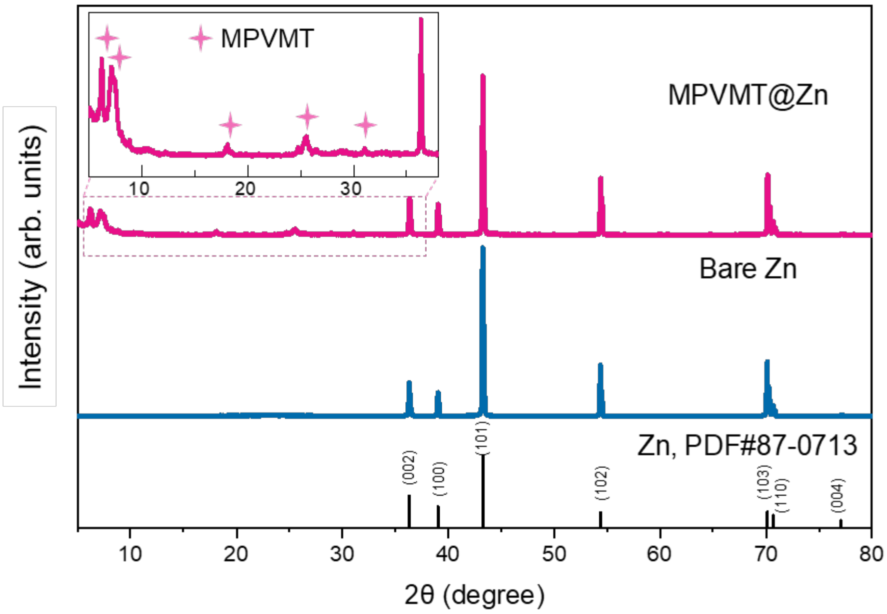

Supplementary Fig. 7 XRD patterns of bare Zn and MPVMT@Zn.

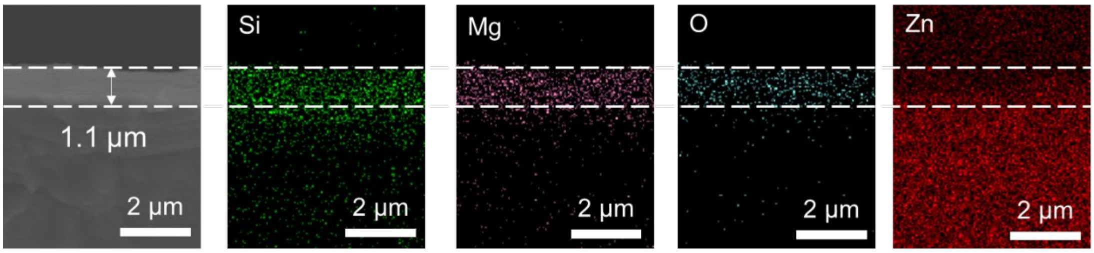

Supplementary Fig. 8 SEM image and corresponding elemental maps of MPVMT@Zn.

Supplementary Note 2. To measure the resistivity of MPVMT coatings, the electrode was individually sandwiched between two stainless steel (SS) blocking electrodes and then the voltage response for the bare Zn electrode and the MPVMT@Zn electrode to a direct current of 2 mA was obtained. The electronic resistivity of MPVMT-coated Zn electrodes was calculated as follows[1]:

$$\rho = \frac{R*S}{L} = \left(\frac{U}{I}\right) * \left(\frac{S}{L}\right)$$

where L is thickness of the coating layer; I is applied current; S is area of the contact between SS and Zn; U is average voltage increase. For the MPVMT coating, the thickness (L) is about 1.1 μm. The contact area (S) is 1.13 cm^2. The average voltage increase is 26.6 mV. Thus, the calculated value of electronic resistivity for MPVMT layers is 1.4×10^5 Ω cm.

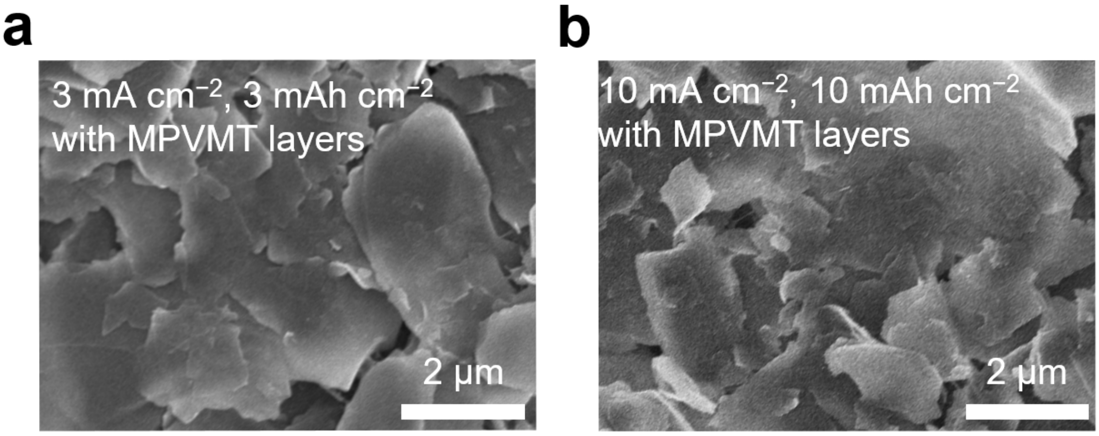

Supplementary Fig. 9 SEM images of MPVMT@Ti after Zn plating at 3 mA cm^{-2}/3 mAh cm^{-2} (**a**) and 10 mA cm^{-2}/10 mAh cm^{-2} (**b**) before removing the MPVMT layers by strong ultrasonication.

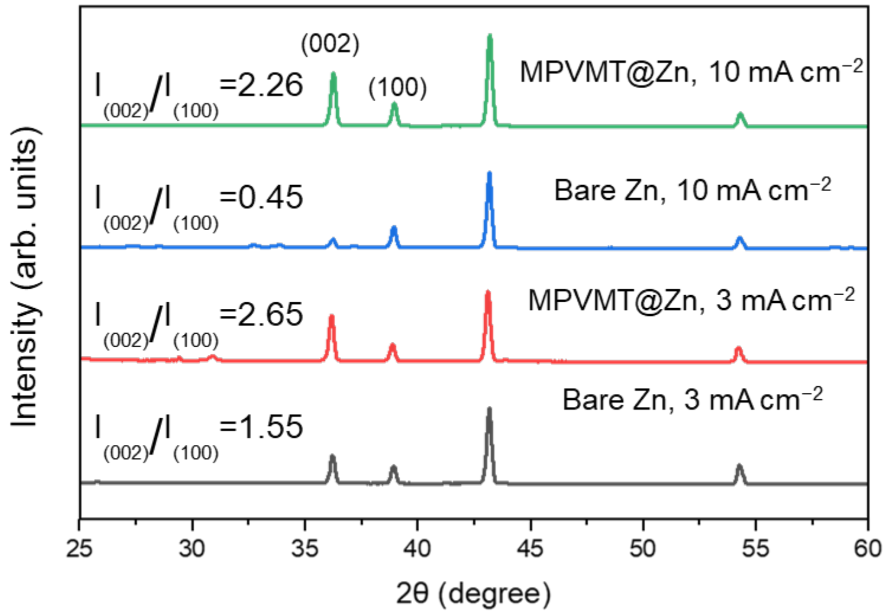

Supplementary Fig. 10 1D XRD patterns of Zn electrodeposits on bare Ti and MPVMT@Ti at 3 mA cm^{-2}/3 mAh cm^{-2} and 10 mA cm^{-2}/10 mAh cm^{-2}.

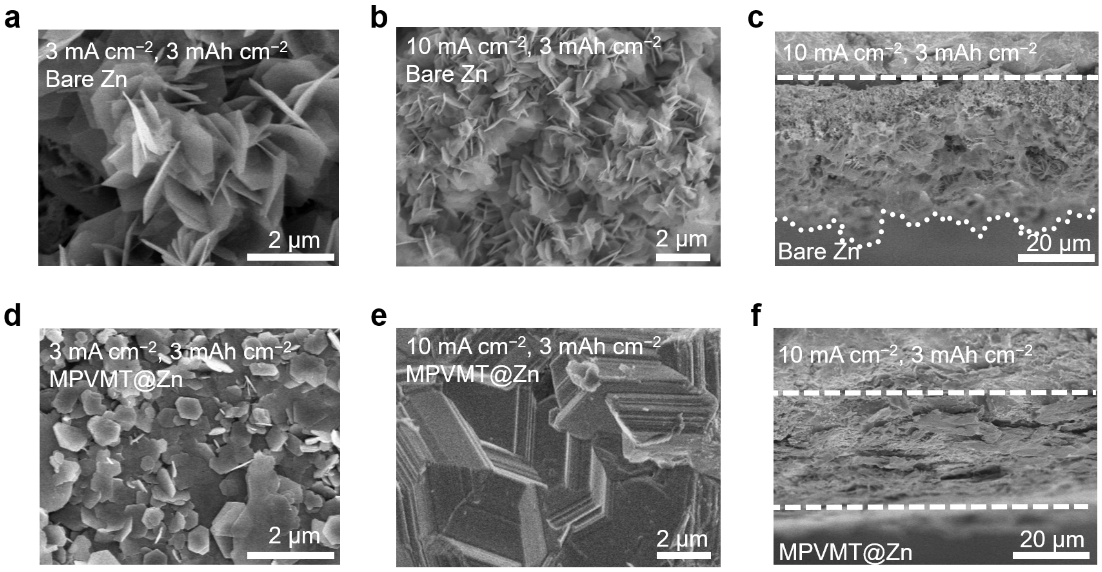

Supplementary Fig. 11 SEM images of Zn electrodeposits on bare Zn at 3 mA cm^{-2}/3 mAh cm^{-2} (**a**), 10 mA cm^{-2}/3 mAh cm^{-2} (**b**) and its cross-sectional image (**c**). SEM images of Zn electrodeposits on MPVMT@Zn at 3 mA cm^{-2}/3 mAh cm^{-2} (**d**), 10 mA cm^{-2}/3 mAh cm^{-2} (**e**) and its cross-sectional image (**f**).

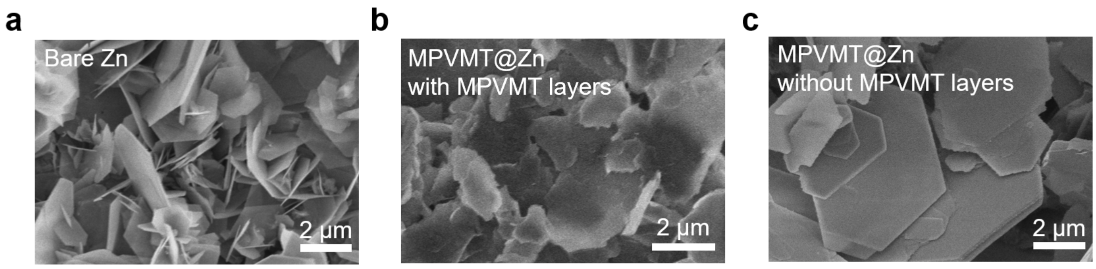

Supplementary Fig. 12 SEM images of surface morphology on bare Zn (**a**), MPVMT@Zn with (**b**) and without MPVMT coatings (**c**) after 200 cycles at 10 mA cm^{-2}/1 mAh cm^{-2}.

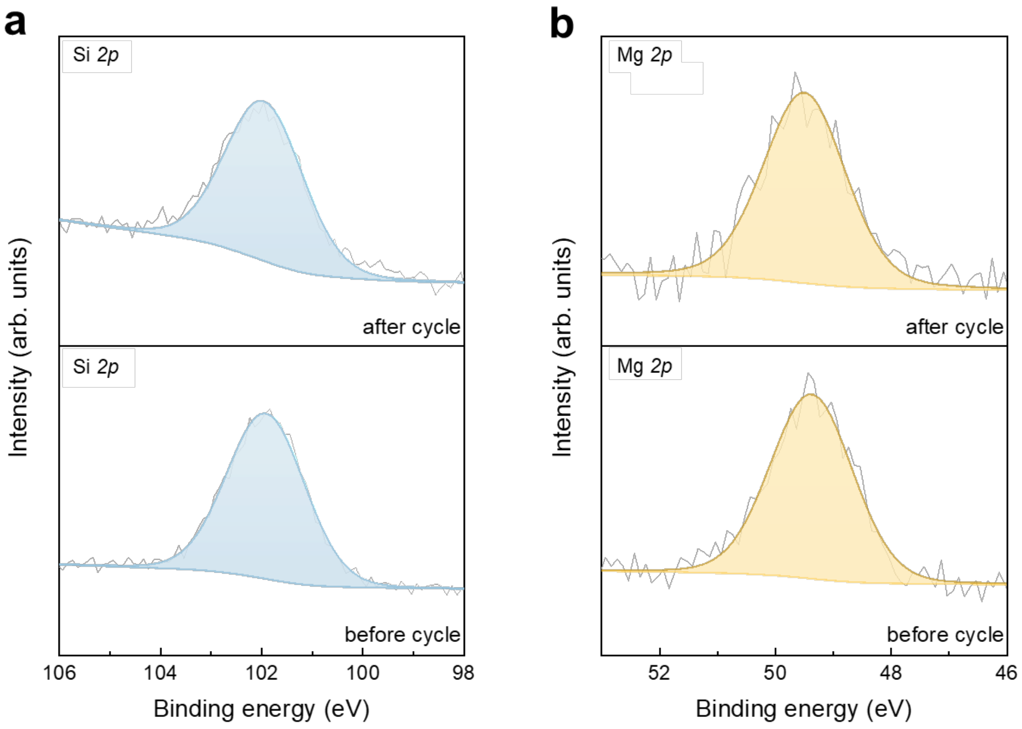

Supplementary Fig. 13 Si *2p* (**a**) and Mg *2p* (**b**) XPS spectra of MPVMT@Zn before and after 200 cycles at 10 mA cm^{-2}/1 mAh cm^{-2}, demonstrating the chemical stability of MPVMT coatings during battery cycling.

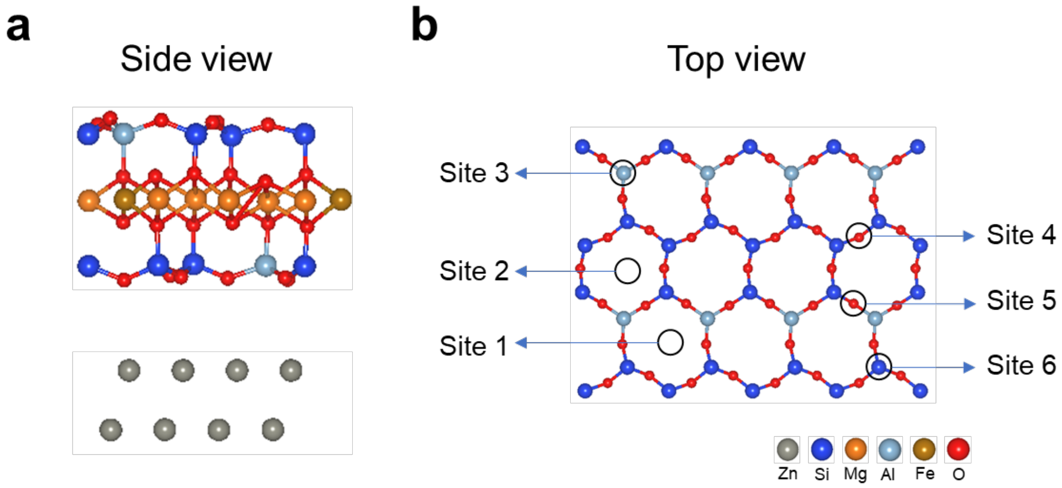

Supplementary Fig. 14 a Constructed DFT models for MPVMT@Zn from the side view. **b** Possibile adsorption sites on the MPVMT.

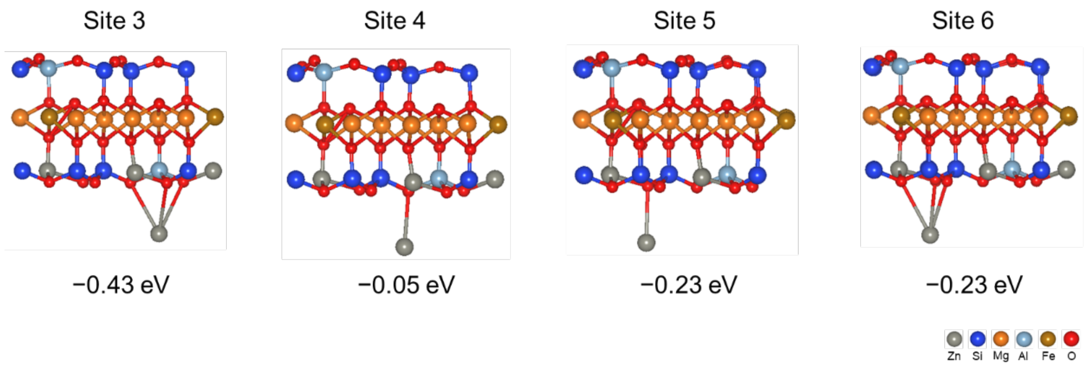

Supplementary Fig. 15 Calculated Zn adsorption energy on the different sites of semi-filled Zn(002)/MPVMT.

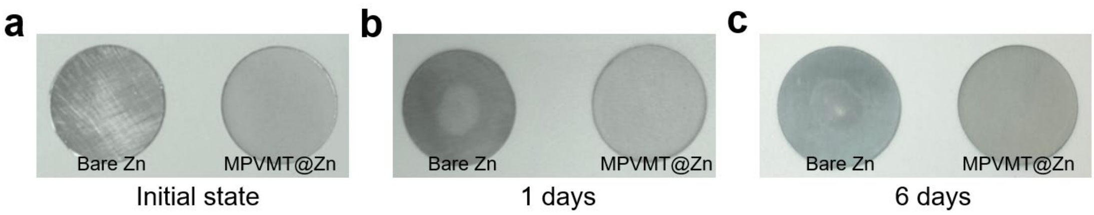

Supplementary Fig. 16 Pictures of bare Zn foil and MPVMT@Zn foil immersed in 2 M $ZnSO_4$ electrolyte for 0 (**a**), 1 (**b**) and 6 days (**c**), respectively.

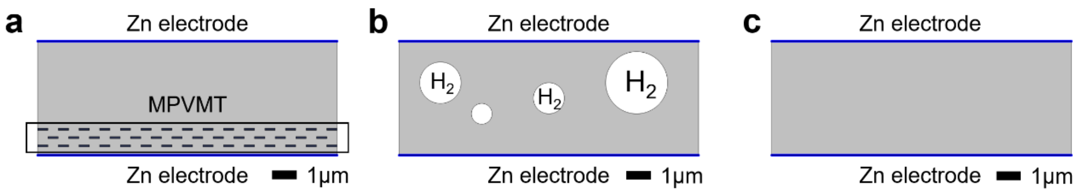

Supplementary Fig. 17 Two-dimensional geometric model for simulating Zn^{2+} deposition for MPVMT@Zn (**a**), bare Zn with severe HER (**b**) and bare Zn without severe HER (**c**).

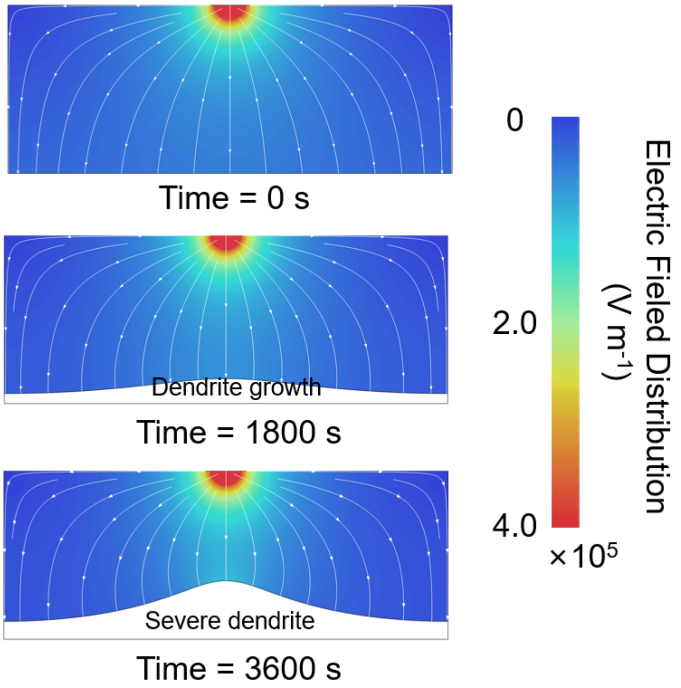

Supplementary Fig. 18 Electric field simulation for bare Zn plating process at the electrolyte/electrode interface without severe HER and interfacial turbulence at 0 s, 1800 s and 3600 s.

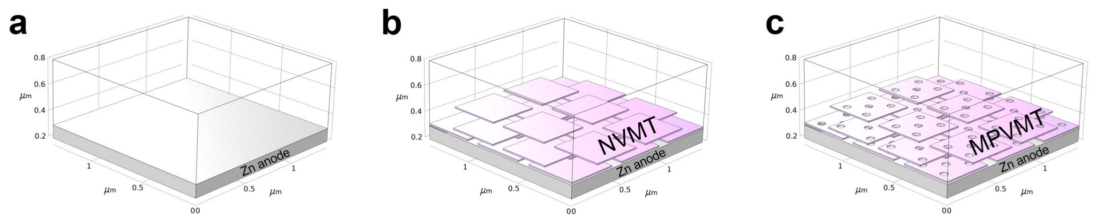

Supplementary Fig. 19 Three-dimensional geometric model for simulating electric field for bare Zn (**a**), NVMT@Zn (**b**) and MPVMT@Zn (**c**).

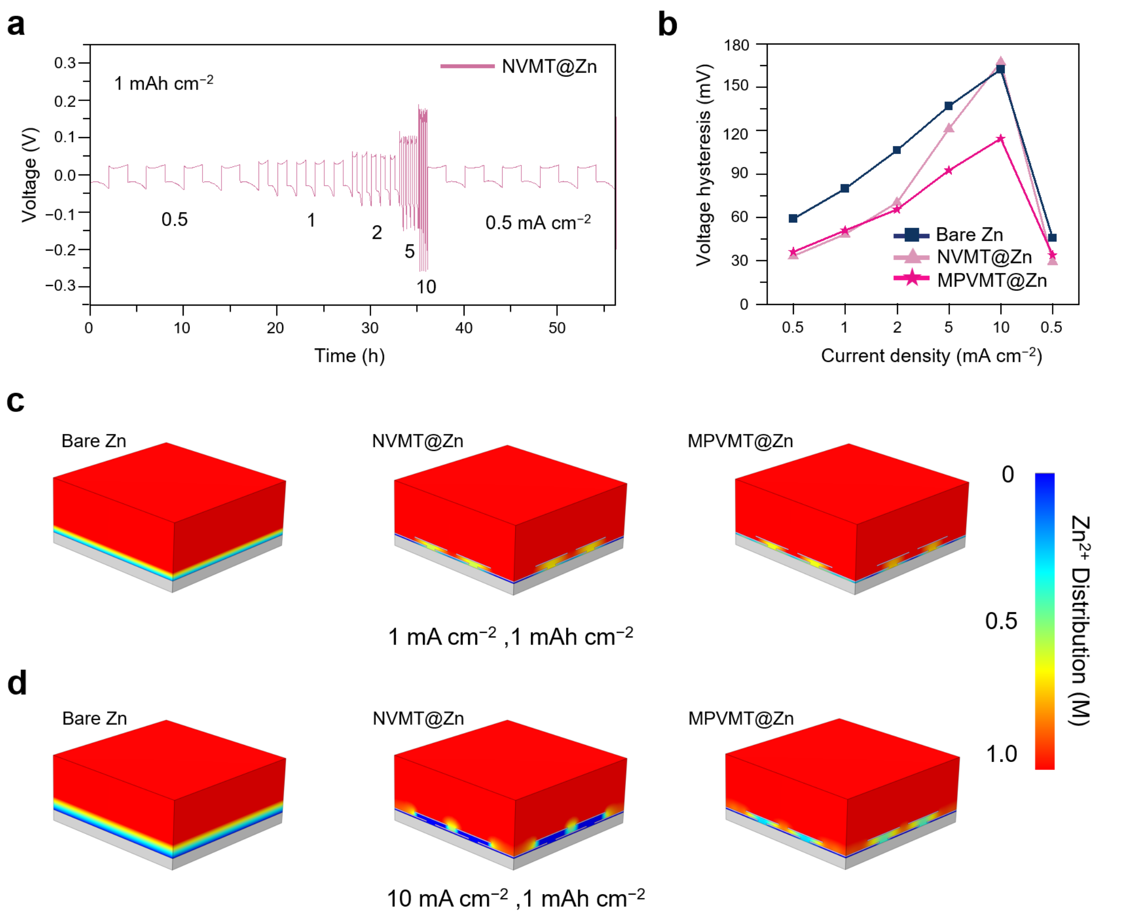

Supplementary Fig. 20 Rate performance of different symmetric cells and corresponding COMSOL simulation. a Rate performance of NVMT@Zn electrodes at different current densities ranging from 0.5 to 10 mA cm^{-2} with the same capacity of 1 mAh cm^{-2}. **b** Corresponding voltage hysteresis of rate performance curves for bare Zn, NVMT@Zn and MPVMT@Zn at different current densities. COMSOL simulation of Zn^{2+} distribution of bare Zn, NVMT@Zn and MPVMT@Zn at 1 mA cm^{-2} (**c**) and 10 mA cm^{-2} (**d**) with the same capacity of 1 mAh cm^{-2}.

Supplementary Note 3. To further understand the Zn transport behavior of bare Zn, NVMT@Zn and MPVMT@Zn, Zn^{2+} distribution was simulated at 1 mA cm^{-2} and 10 mA cm^{-2}. The concentration distribution of Zn^{2+} near the electrode surface reflects the degree of concentration polarization due to the fast or slow ion transport. For bare Zn anodes, significant concentration delamination is observed on the surface at both 1 mA cm^{-2} and 10 mA cm^{-2}, and the Zn^{2+} concentration near the electrode surface significantly decreases at 10 mA cm^{-2}. This is because when the current is raised, the sluggish ion migration rate cannot keep up with the sharply increasing charge transfer

rate, thus forming a distinct concentration gradient near the surface of Zn electrode. However, both NVMT@Zn and MPVMT@Zn have local accelerating electric fields, which can enhance ion transport, and their Zn^{2+} concentration distributions are similar at a small current. Nevertheless, at 10 mA cm^{-2}, because of the flexuose transport path of ions in NVMT layers and the weak acceleration effect, serious concentration polarization occurs on the electrode surface, where the Zn^{2+} concentration near the electrode surface is even lower than that of the bare Zn anode. In contrast, the MPVMT layer, equipped with acceleration channels for ions and abundant local accelerating electric fields around its pore structure, greatly facilitates the rapid transport of Zn^{2+} to the electrode surface at large current densities and reduces voltage hysteresis.

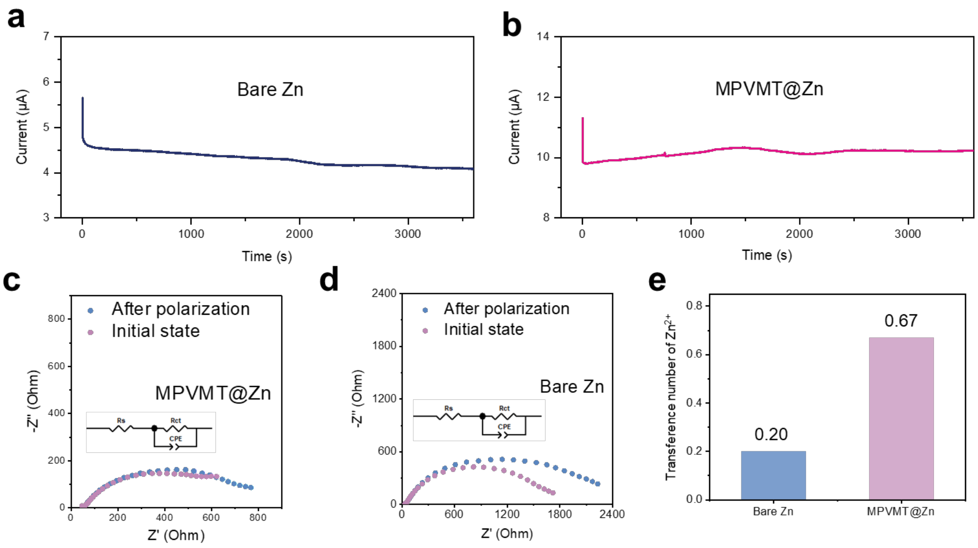

Supplementary Fig. 21 Calculating Zn^{2+} transference number based on CA tests. CA curves of bare Zn (**a**) and MPVMT@Zn (**b**) symmetric cells. Nyquist plots for bare Zn (**c**) and MPVMT@Zn (**d**) symmetric cells before and after polarization. The insets are their fitting equivalent circuit. **e** The calculated transference number of bare Zn and MPVMT@Zn.

Supplementary Note 4. Zn^{2+} transference number of MPVMT@Zn and bare Zn were determined in symmetrical cells by recording the impedance before and after CA tests under a polarization of 10 mV for 3600 s based on the classic Bruce-Vincent method. The transference number (t) is calculated as follows:

$$t=\frac{I_S(\Delta V-I_0R_0)}{I_0(\Delta V-I_SR_S)}$$

$$t_{\mathrm{MPVMT@Zn}}=\frac{9.6\ \mu A\times(10\ mV-11.3\ \mu A\times 635.2\ \Omega)}{11.3\ \mu A\times(10\ mV-9.6\ \mu A\times 669.1\ \Omega)}\approx 0.67$$

$$t_{\mathrm{bare\ Zn}}=\frac{4.1\ \mu A\times(10\ mV-5.7\ \mu A\times 1643\ \Omega)}{5.7\ \mu A\times(10\ mV-4.1\ \mu A\times 1892\ \Omega)}\approx 0.20$$

where ΔV is the applied voltage, I_0 and I_S are the initial and steady current, R_0 and R_S are the initial and steady resistance, respectively.

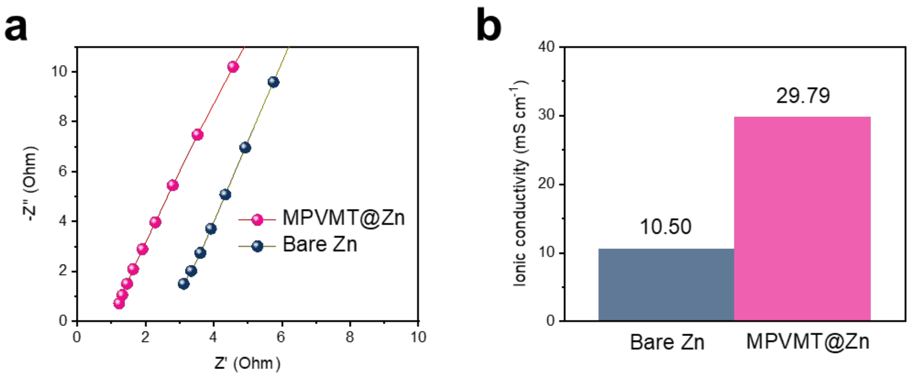

Supplementary Fig. 22 Calculating ionic conductivity based on EIS results. a EIS results of the symmetric cells in SS/MPVMT@Zn/GF or SS/bare Zn/GF configurations (immersed in 2 M $ZnSO_4$). **b** Calculated ionic conductivity based on the EIS results.

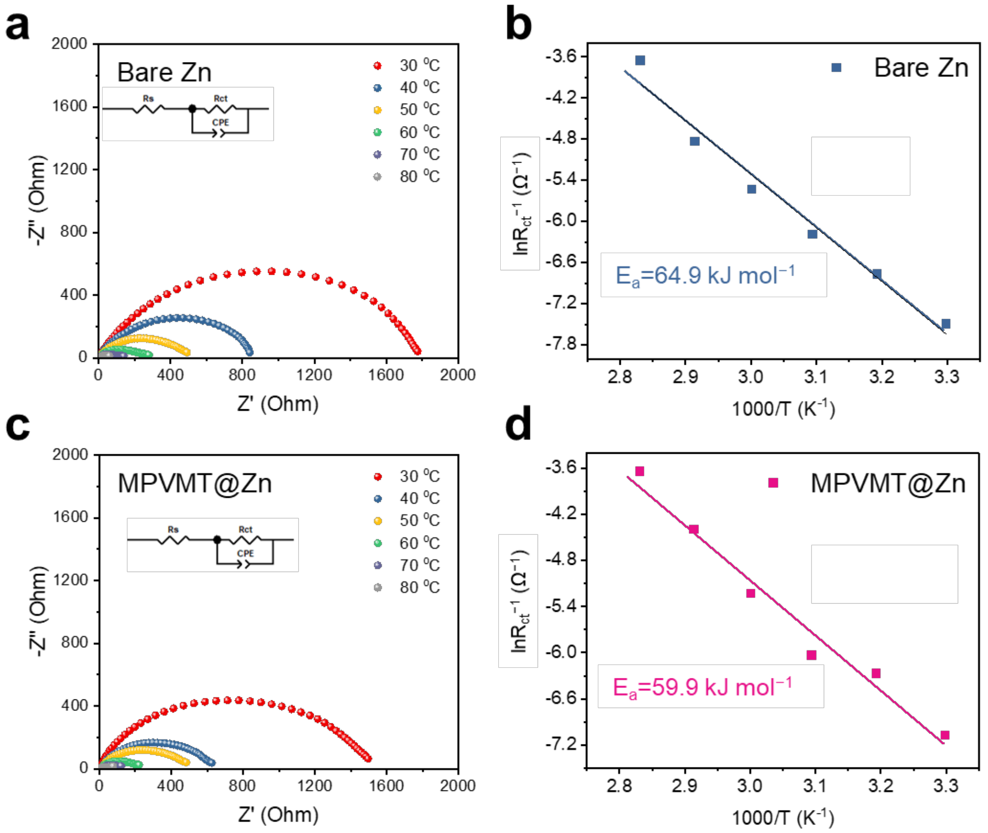

Supplementary Fig. 23 Calculating E_a based on temperature-dependent EIS. Nyquist plots at different temperatures of bare Zn (**a**) and MPVMT@Zn (**c**) symmetrical cells. The insets are their fitting equivalent circuit. Corresponding Arrhenius curves and calculated E_a of Zn (**b**) and MPVMT@Zn (**d**).

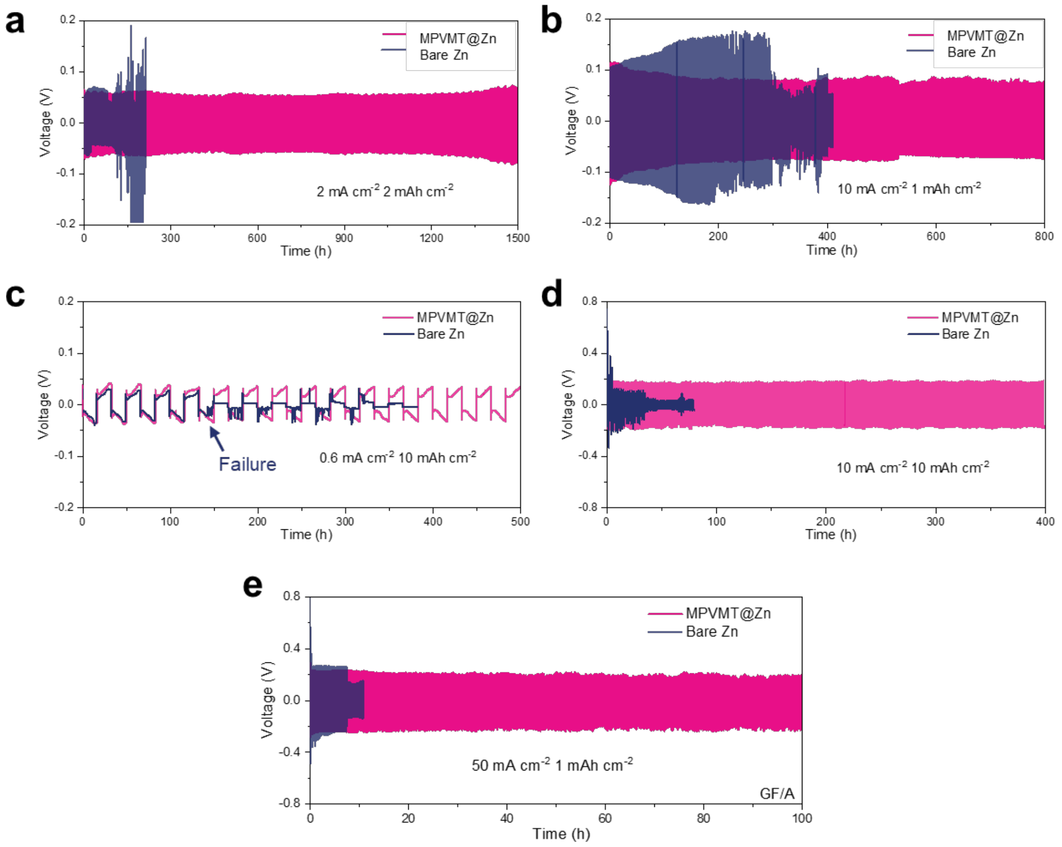

Supplementary Fig. 24 Cycling performance of bare Zn and MPVMT@Zn symmetric cells using GF/D separators at 2 mA cm^{-2}/2 mAh cm^{-2} (**a**), 10 mA cm^{-2}/1 mAh cm^{-2} (**b**), 0.6 mA cm^{-2}/10 mAh cm^{-2} (**c**) and 10 mA cm^{-2}/10 mAh cm^{-2} (**d**), and GF/A separators at 50 mA cm^{-2}/1 mAh cm^{-2} (**e**), respectively. Note that the performance of symmetric cells with thin separators (GF/A) is inferior to that of symmetric cells with thick separators (GF/D).

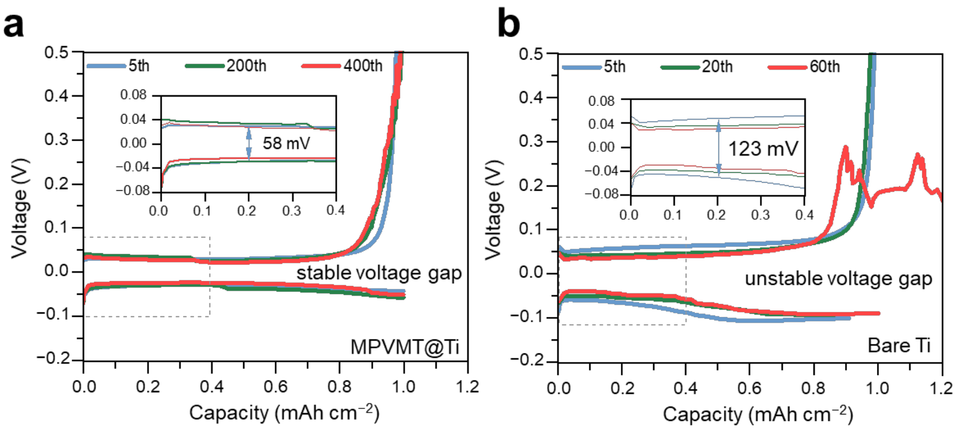

Supplementary Fig. 25 The voltage profiles at different cycles on MPVMT@Ti (**a**) and bare Ti (**b**). The insets are corresponding to enlarged voltage profiles.

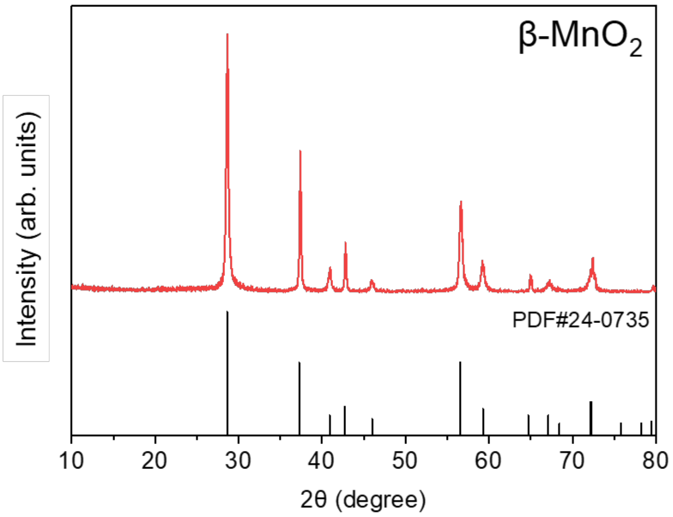

Supplementary Fig. 26 XRD pattern of the as-synthesized β-MnO_2.

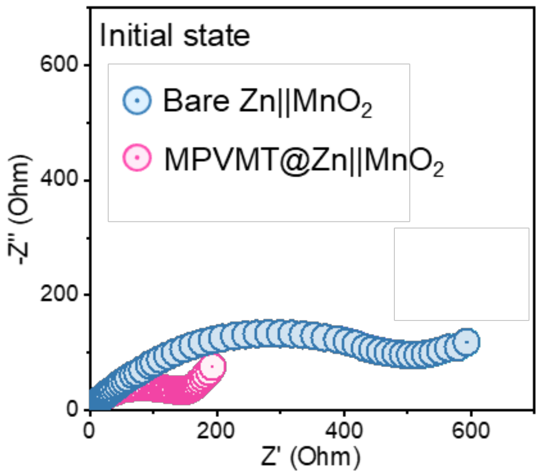

Supplementary Fig. 27 Nyquist plots of Zn||MnO_2 and MPVMT@Zn||MnO_2 full cells at initial state.

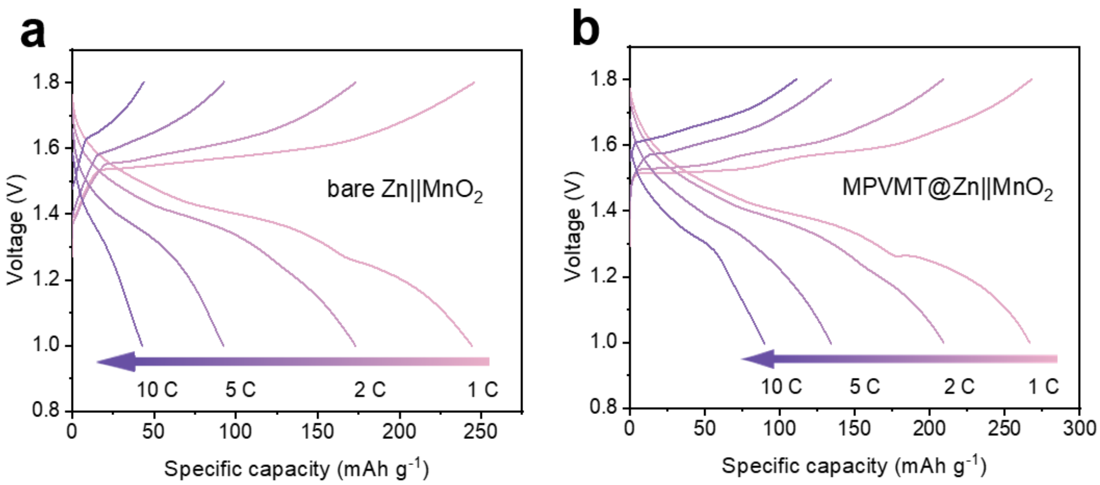

Supplementary Fig. 28 The charge/discharge profiles at different current densities for Zn||MnO_2 (**a**) and MPVMT@Zn||MnO_2 (**b**) liquid full cells with 2 M $ZnSO_4$ and 0.1 M $MnSO_4$ electrolyte.

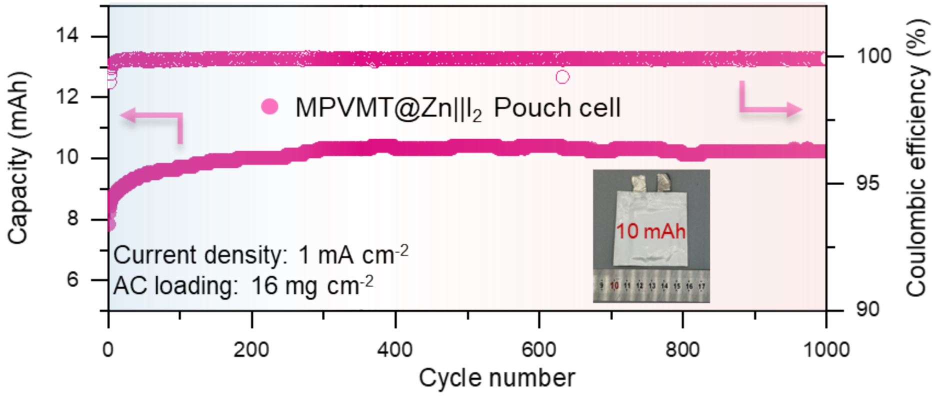

Supplementary Fig. 29 Cycling performance of MPVMT@Zn||I_2 pouch cells at 1 mA cm^{-2}. The inset is the photo of MPVMT@Zn||I_2 pouch cell with the capacity of 10 mAh.

Supplementary Table 1. Eight typical phyllosilicates matching Zn(002) were screened based on $d_{substrate}$: $d_{Zn(002)}$=2:1.

Anode metal	Potential matching substrate	Lattice parameter (Å)	Lattice mismatch δ (%)
Zn(002) (a=b=2.665, 2a=2b=5.33)	Vermiculite (002)	a=5.33, b=5.31	0.38
	Montmorilonite(002)	a=b=5.15	3.38
	Kaolinite(002)	a=5.16, b=5.17	3.19
	Talc(002)	a=b=5.26	1.31
	Serpentine(002)	a=b=5.30	0.56
	Phlogopite(002)	a=b=5.36	0.56
	Illite(002)	a=b=5.18	2.81
	Chlorite(002)	a=b=5.17	3.00

Supplementary Table 2. Comparison of lattice mismatch between recently reported substrates and Zn(002) plane, and their corresponding electrochemical performance.

Substrates	Lattice misfit (%)	Zn asymmetric cell: Current density ($mA\ cm^{-2}$)	Zn asymmetric cell: Coulombic efficiency (%)	Zn symmetric cell: Zn‖Zn Current density ($mA\ cm^{-2}$)	Zn symmetric cell: Time (h)	Zn symmetric cell: Cumulative plating capacity ($Ah\ cm^{-2}$)	Ref.
Graphene	7.41	4 (Zn‖SS)	99%	/	/	/	[2]
MoS_2	21.40	/	/	/	/	/	[3]
Zn_5Cu	3.60	1 (Zn‖Ti)	99.2%	2.5	1600	2	[4]
$ZnAl_2$	6.10	/	/	/	/	/	[4]
Zn_3Mn	2.30	10 (Zn‖Cu)	99.62%	80	750	30	[5]

ZnSe	13.16	2 (Zn||Ti)	99.2%	1	1530	0.765	[6]
				30	170	2.55	
Ag(111)	7.21	/	/	/	/	/	[7]
Sn(002)	~16	1 (Zn||Cu)	98.9%	1	500	0.25	[8]
				2	330	0.33	
In(002)	15.81	/	/	5	400	1	[9]
Vermiculite (002)	0.38	2 (Zn||Ti)	99.4%	1	2000	1	This work
				2	1500	1.5	
				5	680	1.7	
				10	800	4	
				50	300	7.5	

Supplementary Table 3. Corresponding average atomic fractions of EDS maps.

Element	Family	Atomic Fraction (%)	Atomic Error (%)	Mass Fraction (%)	Mass Error (%)	Fit error (%)
O	K	64.99	15.54	50.79	8.79	0.23
Si	K	15.47	3.63	21.23	3.57	1.87
Mg	K	11.48	2.74	13.62	2.35	2.04
Al	K	4.45	1.06	5.87	1.01	1.08
Fe	K	1.96	0.39	5.32	0.68	0.55
K	K	1.66	0.37	3.17	0.5	1.47

Supplementary Table 4. Fitting results for MPVMT@Zn and bare Zn symmetric cells at different temperatures.

Symmetric cells	Resistance	30 ºC	40 ºC	50 ºC	60 ºC	70 ºC	80 ºC
Bare Zn	R_{ct} (Ω)	1804	870.5	486.7	252.4	125.6	38.77
MPVMT@Zn	R_{ct} (Ω)	1187	531	417.4	186.6	81.12	38.06

Supplementary Table 5. Summary of electrochemical performance of recently typical Zn anode with different strategies.

Strategies	Current density (mA cm^{-2})	Time (h)	Cumulative plating capacity (Ah cm^{-2})	Ref.
MPVMT	1	2000	1	This work
	2	1500	1.5	
	5	680	1.7	
	10	800	4	
	50	300	7.5	
PVDF-Sn	1	1200	0.6	[10]
	5	500	1.25	
Sn@NHCF	1	370	0.185	[11]
ZF@C-TiO_2	2	280	0.28	[12]
AAn-COF	1	300	0.15	[13]
	5	400	1	
	10	300	1.5	
	20	300	3	
ZSO	10	750	3.75	[14]
TiN	2	1000	1	[15]

	5	500	1.25	
FCOF	8	750	3	16
	5	500	1.25	
3D-ZGCF	10	400	2	17
	20	150	1.5	
PCu	1	200	0.1	18
	10	200	1	
3D VG	5	75	0.1875	19
	10	600	3	
MGA	20	28	0.28	20
	1	400	0.2	
P-MIEC	2	220	0.22	21
	5	180	0.45	
	10	100	0.5	
La^{3+}-ZS	1	1200	0.6	22
	10	160	0.8	
Saccharin	10	550	2.75	23
	40	220	4.4	
I^{-}	1	600	0.3	24

Zn_5Cu	2.5	1600	2	[4]
GFA	3	700	1.05	[1]
PS-Zn	10	500	2.5	[25]

Supplementary Table 6. Performance comparison of Zn||MnO_2 liquid coin cells from previous researches with different modified Zn anodes and this work.

Anode	Mass loading (mg cm^{-2})	Current Density (A g^{-1})	Capacity retention improvement rate	Ref.
MPVMT	1~2	0.616	217% (500 cycles)	This work
PS-Zn	1.4	0.5	104% (200 cycles)	25
Zn-P-MIEC	1	0.5	125% (500 cycles)	21
Zn@In	/	1	239% (500 cycles)	26
GBL additive	/	0.5	134% (400 cycles)	27
β-CD additive	/	1	165% (500 cycles)	28
ZF@F-TiO_2	/	1	112% (300 cycles)	12
Zn@PFSA	/	0.616	230% (500 cycles)	29
PVDF-Sn@Zn	0.8	2	171% (500 cycles)	10
Zn-AAn-COF	1.3~1.5	1	151% (500 cycles)	13
TiN(200)@Zn	~2	0. 370	141% (500 cycles)	15
PVA@SR-$ZnMoO_4$	1.5	1	127% (500 cycles)	30

Capacity retention improvement rate is defined as follows:

$$\text{Capacity retention improvement rate}=\frac{\text{Cycled Capacity}_{\text{modified Zn}}/\text{Initial Capacity}_{\text{modified Zn}}}{\text{Cycled Capacity}_{\text{bare Zn}}/\text{Initial Capacity}_{\text{bare Zn}}}$$

Supplementary Table 7. Comparison of the MPVMT@Zn||MnO_2 pouch cell with previously reported Zn metal pouch cells.

Electrodes	Electrolyte	Total capacity (mAh)	Mass loading (mg cm^{-2})	Ref.
MPVMT@ Zn||MnO_2	2 M $ZnSO_4$ +0.1 M $MnSO_4$	1250	13.5	This work
PVDF-Sn@ Zn||MnO_2@C	2 M $ZnSO_4$ +0.1 M $MnSO_4$	20	0.8	10
GFA@ Zn||I_2	1 M $ZnSO_4$	160	15	1
Zn||ZVO	2 M $ZnSO_4$ in CarraChi gel	900	15	31
Zn||$Zn_{0.25}V_2O_5$ •nH_2O	RME	1000	20	32
Zn||KVOH	TMP-40	9	2.1	33
Zn||PANI	1 M $Zn(PS)_2$ + 0.2 TBATS	7	2	34
Zn||MnO_2	3 M $Zn(OTf)_2$ +0.1 M $Mn(OTf)_2$	1550	12	35
NGO@Zn||Li Mn_2O_4	2 M Li_2SO_4 +1 M $ZnSO_4$	32	16.7	36
Zn||$VOPO_4$	4 M $Zn(OTf)_2$ +0.5 M Me3EtNOTf	50	3	37

Supplementary Table 8. The parameters of every component in the ampere-hour MPVMT@Zn||MnO_2 pouch cell.

Components	Length (mm)	Width (mm)	Thickness (mm)	Weight (g)
Anode (Zn foil)	150	130	0.05*2	6.9*2
Cathode (MnO_2)	150	130	0.15*2	2.6*2
Separator (GF)	160	140	0.27*2	2.7*2
Current collector (Ti foil)	150	130	0.02	1.7
Electrolyte (2 M $ZnSO_4$+0.1 M $MnSO_4$)	/	/	/	48.8 (36 ml)
Aluminum-plastic film	330	150	0.113*2	8.6

Supplementary Note 5. The volumetric energy density ($E_{volumetric}$) of the pouch cell based on all components (listed in Supplementary Table 8) except electrolytes, which accounts for the diffusion of the electrolyte within cathodes and separators resulting in little contribution to the overall volume, is calculated as follows:

$$E_{volumetric} = \frac{1.74\times10^{6}}{150 \times 130\times(0.05\times2+0.15\times2+0.27\times2+0.02+0.113\times2)}$$

$$\approx 75 \text{ Wh L}^{-1}$$

The specific energy density ($E_{specific}$) of the pouch cell is calculated as follows:

$$E_{specific} \text{ (active materials)} = \frac{1.74\times10^{3}}{(6.9\times2+2.6\times2)} \approx 92 \text{ Wh kg}^{-1},$$

and

$$E_{specific} \text{ (whole cell)} = \frac{1.74\times10^{3}}{(6.9\times2+2.6\times2+2.7\times2+1.7+48.8+8.6)}$$

$$\approx 21 \text{ Wh kg}^{-1}$$

Supplementary References

1 Liang, G. *et al.* Gradient fluorinated alloy to enable highly reversible Zn-metal anode chemistry. *Energy Environ. Sci.* **15**, 1086-1096 (2022).

2 Zheng, J. *et al.* Reversible epitaxial electrodeposition of metals in battery anodes. *Science* **366**, 645-648 (2019).

3 Wang, Y. *et al.* MoS_2-Mediated Epitaxial Plating of Zn Metal Anodes. *Adv. Mater.* **35**, e2208171 (2022).

4 Ji, J. *et al.* Zinc-Contained Alloy as a Robustly Adhered Interfacial Lattice Locking Layer for Planar and Stable Zinc Electrodeposition. *Adv. Mater.* **35**, e2211961 (2023).

5 Tian, H. *et al.* Stable, high-performance, dendrite-free, seawater-based aqueous batteries. *Nat. Commun.* **12**, 237 (2021).

6 Yang, X. *et al.* Interfacial Manipulation via In Situ Grown ZnSe Cultivator toward Highly Reversible Zn Metal Anodes. *Adv. Mater.* **33**, e2105951 (2021).

7 Yi, Z. *et al.* An Ultrahigh Rate and Stable Zinc Anode by Facet-Matching-Induced Dendrite Regulation. *Adv. Mater.* **34**, e2203835 (2022).

8 Li, S. *et al.* Toward Planar and Dendrite-Free Zn Electrodepositions by Regulating Sn-Crystal Textured Surface. *Adv. Mater.* **33**, e2008424 (2021).

9 Ouyang, K. *et al.* A New Insight into Ultrastable Zn Metal Batteries Enabled by In Situ Built Multifunctional Metallic Interphase. *Adv. Funct. Mater.* **32**, 2109749 (2021).

10 Cao, Q. *et al.* Gradient design of imprinted anode for stable Zn-ion batteries. *Nat. Commun.* **14**, 641 (2023).

11 Yu, H. *et al.* Confining Sn nanoparticles in interconnected N-doped hollow carbon spheres as hierarchical zincophilic fibers for dendrite-free Zn metal anodes. *Sci. Adv.* **8**, eabm5766 (2022).

12 Zhang, Q. *et al.* Revealing the role of crystal orientation of protective layers for stable zinc anode. *Nat. Commun.* **11**, 3961 (2020).

13 Guo, C. *et al.* Synergistic Manipulation of Hydrogen Evolution and Zinc Ion Flux in Metal-Covalent Organic Frameworks for Dendrite-free Zn-based Aqueous Batteries. *Angew. Chem. Int. Ed.* **61**, e202210871 (2022).

14 Peng, H. *et al.* Constructing fast-ion-conductive disordered interphase for high-performance zinc-ion and zinc-iodine batteries. *Matter* **5**, 1-16 (2022).

15 Zheng, J. *et al.* Preferred Orientation of TiN Coatings Enables Stable Zinc Anodes. *ACS Energy Lett.* **7**, 197-203 (2021).

16 Zhao, Z. *et al.* Horizontally arranged zinc platelet electrodeposits modulated by fluorinated covalent organic framework film for high-rate and durable aqueous zinc ion batteries. *Nat. Commun.* **12**, 6606 (2021).

17 Xue, P. *et al.* A MOF-Derivative Decorated Hierarchical Porous Host Enabling Ultrahigh Rates and Superior Long-Term Cycling of Dendrite-Free Zn Metal Anodes. *Adv. Mater.* **34**, e2110047 (2022).

18 Zhou, J. *et al.* Establishing Thermal Infusion Method for Stable Zinc Metal

Anodes in Aqueous Zinc-Ion Batteries. *Adv. Mater.* **34**, e2200782 (2022).
19 Li, C. *et al.* Directly Grown Vertical Graphene Carpets as Janus Separators toward Stabilized Zn Metal Anodes. *Adv. Mater.* **32**, e2003425 (2020).
20 Zhou, J. *et al.* Encapsulation of Metallic Zn in a Hybrid MXene/Graphene Aerogel as a Stable Zn Anode for Foldable Zn-Ion Batteries. *Adv. Mater.* **34**, e2106897 (2022).
21 Zhang, M. *et al.* Construction of mixed ionic-electronic conducting scaffolds in Zn powder: A scalable route to dendrite-free and flexible Zn anodes. *Adv. Mater.* **34**, e2200860 (2022).
22 Zhao, R. *et al.* Lanthanum nitrate as aqueous electrolyte additive for favourable zinc metal electrodeposition. *Nat. Commun.* **13**, 3252 (2022).
23 Huang, C. *et al.* Stabilizing Zinc Anodes by Regulating the Electrical Double Layer with Saccharin Anions. *Adv. Mater.* **33**, e2100445 (2021).
24 Zhang, Q. *et al.* Halogenated Zn^{2+} Solvation Structure for Reversible Zn Metal Batteries. *J. Am. Chem. Soc.* **144**, 18435-18443 (2022).
25 Li, Q. *et al.* Tailoring the metal electrode morphology via electrochemical protocol optimization for long-lasting aqueous zinc batteries. *Nat. Commun.* **13**, 3699 (2022).
26 Xiao, P. *et al.* An anticorrosive zinc metal anode with ultra-long cycle life over one year. *Energy Environ. Sci.* **15**, 1638-1646 (2022).
27 Huang, H. *et al.* Boosting Reversibility and Stability of Zn Anodes via Manipulation of Electrolyte Structure and Interface with Addition of Trace Organic Molecules. *Adv. Energy Mater.* **12**, 2202419 (2022).
28 Qiu, M. *et al.* Anion-Trap Engineering toward Remarkable Crystallographic Reorientation and Efficient Cation Migration of Zn Ion Batteries. *Angew. Chem. Int. Ed.* **61**, e202210979 (2022).
29 Hong, L. *et al.* Highly Reversible Zinc Anode Enabled by a Cation-Exchange Coating with Zn-Ion Selective Channels. *ACS Nano* **16**, 6906-6915 (2022).
30 Chen, A. S. *et al.* Multifunctional SEI-like structure coating stabilizing Zn anodes at a large current and capacity. *Energy Environ. Sci.* **16**, 275-284 (2023).
31 Wang, F. *et al.* Production of gas-releasing electrolyte-replenishing Ah-scale zinc metal pouch cells with aqueous gel electrolyte. *Nat. Commun.* **14**, 4211 (2023).
32 Wang, Y. *et al.* Sulfolane-containing aqueous electrolyte solutions for producing efficient ampere-hour-level zinc metal battery pouch cells. *Nat. Commun.* **14**, 1828 (2023).
33 Wang, W. *et al.* Regulating interfacial reaction through electrolyte chemistry enables gradient interphase for low-temperature zinc metal batteries. *Nat. Commun.* **14**, 5443 (2023).
34 Chen, S. *et al.* Coordination modulation of hydrated zinc ions to enhance redox reversibility of zinc batteries. *Nat. Commun.* **14**, 3526 (2023).
35 Zhang, N. *et al.* Rechargeable aqueous zinc-manganese dioxide batteries with high energy and power densities. *Nat. Commun.* **8**, 405 (2017).
36 Zhou, J. *et al.* Ultrathin Surface Coating of Nitrogen-Doped Graphene Enables

Stable Zinc Anodes for Aqueous Zinc-Ion Batteries. *Adv. Mater.* **33**, 2101649 (2021).
37 Cao, L. *et al.* Fluorinated interphase enables reversible aqueous zinc battery chemistries. *Nat. Nanotechnol.* **16**, 902 (2021).
