# Supplementary material for: An extended substrate screening strategy enabling a low lattice mismatch for highly reversible zinc anodes
Source: Nat Commun. 2024 Jan 25;15:753. doi: 10.1038/s41467-024-44893-0 (PMC10810881; doi:10.1038/s41467-024-44893-0)
Supplement: Supplementary file 3 — Reporting Summary [file 41467_2024_44893_MOESM3_ESM.pdf]

nature portfolio

Corresponding author(s): Guangmin Zhou

Last updated by author(s): Dec 26, 2023

Reporting Summary

Nature Portfolio wishes to improve the reproducibility of the work that we publish. This form provides structure for consistency and transparency in reporting. For further information on Nature Portfolio policies, see our Editorial Policies and the Editorial Policy Checklist.

Statistics

For all statistical analyses, confirm that the following items are present in the figure legend, table legend, main text, or Methods section.

n/a	Confirmed	
☐	☒	The exact sample size (*n*) for each experimental group/condition, given as a discrete number and unit of measurement
☐	☒	A statement on whether measurements were taken from distinct samples or whether the same sample was measured repeatedly
☒	☐	The statistical test(s) used AND whether they are one- or two-sided<br>*Only common tests should be described solely by name; describe more complex techniques in the Methods section.*
☒	☐	A description of all covariates tested
☒	☐	A description of any assumptions or corrections, such as tests of normality and adjustment for multiple comparisons
☐	☒	A full description of the statistical parameters including central tendency (e.g. means) or other basic estimates (e.g. regression coefficient) AND variation (e.g. standard deviation) or associated estimates of uncertainty (e.g. confidence intervals)
☒	☐	For null hypothesis testing, the test statistic (e.g. F, t, r) with confidence intervals, effect sizes, degrees of freedom and P value noted<br>*Give P values as exact values whenever suitable.*
☒	☐	For Bayesian analysis, information on the choice of priors and Markov chain Monte Carlo settings
☒	☐	For hierarchical and complex designs, identification of the appropriate level for tests and full reporting of outcomes
☒	☐	Estimates of effect sizes (e.g. Cohen's d, Pearson's r), indicating how they were calculated

Our web collection on statistics for biologists contains articles on many of the points above.

Software and code

Policy information about availability of computer code

Data collection	The electrochemical data were collected by the “LAND CT2001A”, "Neware CT-4008T", "CHI760E" and "Malvern Zetasizer Nano S90" system. Crystallographic phase and chemical composition data were investigated by the "Bruker D8 Advance" and "PHI 5000 Versa Probe II" system. Surface characteristic and microstructure were characterized by the “Ossila Contact angle”, "Hitachi SU8010", "FEI Tecani F30" and "Thermo Fisher Scientific Spectra 300" system. Operando visualization and hydrogen collection was carried out using the "OLYMPUS BX53M" and "ZhongJiaoJinYuan GC7920" system, respectively.
Data analysis	The electrochemical data were analyzed by the "LANDdt V7.4", "BTSDA 8.0.0.471", and "CHI760E". Crystallographic phase and chemical composition data were processed using the "MDI Jade 9" and "Multipak". Surface characteristic and microstructure were investigated by the “Ossila Contact angle”, "NanoScope Analysis 1.9" and "Digital Micrograph GMS3". Density functional theory calculations were performed by the "Vienna Ab-initio Simulation Package 5.4.4". The finite element simulations were conducted using the "COMSOL Multiphysics 6.0". "Origin 2021" and "Microsoft PowerPoint 2019" were used for creating and organizing the graphs.

For manuscripts utilizing custom algorithms or software that are central to the research but not yet described in published literature, software must be made available to editors and reviewers. We strongly encourage code deposition in a community repository (e.g. GitHub). See the Nature Portfolio guidelines for submitting code & software for further information.

Data

Policy information about availability of data

All manuscripts must include a data availability statement. This statement should provide the following information, where applicable:

- Accession codes, unique identifiers, or web links for publicly available datasets
- A description of any restrictions on data availability
- For clinical datasets or third party data, please ensure that the statement adheres to our policy

All data that support the findings of this study are presented in the Manuscript and Supplementary Information, or are available from the corresponding author upon reasonable request. Source data are provided with this paper.

Research involving human participants, their data, or biological material

Policy information about studies with human participants or human data. See also policy information about sex, gender (identity/presentation), and sexual orientation and race, ethnicity and racism.

Reporting on sex and gender	There are no human research participants in our research.
Reporting on race, ethnicity, or other socially relevant groupings	There are no socially relevant groupings in our research.
Population characteristics	There are no population characteristics in our research.
Recruitment	There is no recruitment in our research.
Ethics oversight	There is no ethics oversight in our research.

Note that full information on the approval of the study protocol must also be provided in the manuscript.

Field-specific reporting

Please select the one below that is the best fit for your research. If you are not sure, read the appropriate sections before making your selection.

☐ Life sciences ☐ Behavioural & social sciences ☒ Ecological, evolutionary & environmental sciences

For a reference copy of the document with all sections, see nature.com/documents/nr-reporting-summary-flat.pdf

Ecological, evolutionary & environmental sciences study design

All studies must disclose on these points even when the disclosure is negative.

Study description	We propose the extended substrate screening strategy for stabilizing Zn anodes and verify its availability (dsubstrate: dZn(002)=1:1→dsubstrate: dZn(002)=n:1, n=1, 2). From a series of calculated phyllosilicates satisfying dsubstrate≈2dZn(002), we select vermiculite, which has the lowest lattice mismatch (δ=0.38%) reported so far, as the model to confirm the effectiveness of “2dZn(002)” substrates for Zn anodes protection. Then, we develop a monolayer porous vermiculite (MPVMT) through a large-scale and green preparation as a functional coating for Zn electrodes. Unique “planting Zn(002) seeds” mechanism for “2dZn(002)” substrates is revealed to induce the oriented growth of Zn deposits. Additionally, MPVMT coatings effectively inhibit side reactions and promote Zn2+ transport. This work extends SSS and advances the understanding of Zn nucleation mechanism, paving the way for realizing high-rate and stable Zn-metal batteries.
Research sample	Monolayer porous vermiculite coatings with an ultralow lattice misfit with Zn(002) facet
Sampling strategy	Based on the proposed “extended substrate screening strategy”, we discovered a series of phyllosilicates matching dsubstrate: dZn(002)=2:1 and further selected insulting vermiculite substrates with the lowest lattice mismatch as the model to study characteristics of “2dZn(002)” substrates and their effects on Zn anodes. Additionally, to facilitate zinc ions transport, we prepared a monolayer porous vermiculite (MPVMT) coatings with acceleration channels. Thus, MPVMT coatings enable horizontal growth of Zn electrodeposits without dendrite formation, inhibition of side reactions and fast ion transport.
Data collection	G.Z. conceived the project. Z.Z., L.D., X.X. and H.L. synthesized the materials. Z.Z. and X. Z. carried out the materials characterization and analyzed the data. Q.Z. and M.Z. conducted theoretical simulations. J.X., M.J., B.W., Y.J. and R.M. provided important experimental insights. G.Z. supervised the research and revised the manuscript. All the authors discussed the results and contributed to writing the manuscript.
Timing and spatial scale	From 2022 to 2023. Data collection commences from the initial stage of electrochemical testing and concludes upon the completion of the testing process.

Data exclusions	No data were excluded.
Reproducibility	All experiments were repeated at least three times.
Randomization	This study does not involve quantitative methods that would require randomization.
Blinding	There is no blinding in our research.

Did the study involve field work? ☐ Yes ☒ No

Reporting for specific materials, systems and methods

We require information from authors about some types of materials, experimental systems and methods used in many studies. Here, indicate whether each material, system or method listed is relevant to your study. If you are not sure if a list item applies to your research, read the appropriate section before selecting a response.

Materials & experimental systems

n/a	Involved in the study	
☒	☐	Antibodies
☒	☐	Eukaryotic cell lines
☒	☐	Palaeontology and archaeology
☒	☐	Animals and other organisms
☒	☐	Clinical data
☒	☐	Dual use research of concern
☒	☐	Plants

Methods

n/a	Involved in the study	
☒	☐	ChIP-seq
☒	☐	Flow cytometry
☒	☐	MRI-based neuroimaging
